# The Association of an SNP in the *EXOC4* Gene and Reproductive Traits Suggests Its Use as a Breeding Marker in Pigs

**DOI:** 10.3390/ani11020521

**Published:** 2021-02-17

**Authors:** Yingting He, Xiaofeng Zhou, Rongrong Zheng, Yao Jiang, Zhixiang Yao, Xilong Wang, Zhe Zhang, Hao Zhang, Jiaqi Li, Xiaolong Yuan

**Affiliations:** 1 Guangdong Provincial Key Lab of Agro-Animal Genomics and Molecular Breeding, National Engineering Research Center for Breeding Swine Industry, College of Animal Science, South China Agricultural University, Guangzhou 510642, China; 15521059247@163.com (Y.H.); zxf93715@163.com (X.Z.); 18819266704zrr@sina.com (R.Z.); jyao192@163.com (Y.J.); zhezhang@scau.edu.cn (Z.Z.); zhanghao@scau.edu.cn (H.Z.); 2 Guangdong Dexing Food Co., Ltd., Shantou 515100, China; yao.zx@dexing1996.com; 3 Guangdong Provincial Key Laboratory of Laboratory Animals, Guangdong Laboratory Animals Monitoring Institute, Guangzhou 510260, China; wangxilonggd@163.com

**Keywords:** commercial pig, reproductive traits, gene polymorphism, promoter

## Abstract

**Simple Summary:**

The exocyst complex component 4 (*EXOC4*) gene is essential for the growth and development of humans and animals. However, the molecular mechanisms between *EXOC4* and the reproductive performance of pigs is yet to be elucidated. The findings of our study revealed that single-nucleotide polymorphism (SNP) rs81471943 (C/T) was located in the *EXOC4* gene by Sanger sequencing and PCR-restriction fragment length polymorphism (PCR-RFLP). We then analyzed the relationship between this SNP in *EXOC4* and reproductive traits. We found that CC was the most frequent genotype on number of piglets born alive (NBA), litter weight at birth (LWB), number of piglets weaned (NW), and litter weight at weaning (LWW). Finally, 5′-deletion and luciferase assays showed a positive transcription regulatory element in the *EXOC4* promoter. Therefore, we predicted that −1781G/A might regulate the expression of *EXOC4* by affecting the potential binding of transcription factors *P53*, E26 transformation specific sequence -like 1 transcription factor (*ELK1*), or myeloid zinc finger 1 (*MZF1*).

**Abstract:**

In mammals, the exocyst complex component 4 (*EXOC4*) gene has often been reported to be involved in vesicle transport. The SNP rs81471943 (C/T) is located in the intron of porcine *EXOC4*, while six quantitative trait loci (QTL) within 5–10 Mb around *EXOC4* are associated with ovary weight, teat number, total offspring born alive, and corpus luteum number. However, the molecular mechanisms between *EXOC4* and the reproductive performance of pigs remains to be elucidated. In this study, rs81471943 was genotyped from a total of 994 Duroc sows, and the genotype and allele frequency of SNP rs81471943 (C/T) were statistically analyzed. Then, the associations between SNP rs81471943 and four reproductive traits, including number of piglets born alive (NBA), litter weight at birth (LWB), number of piglets weaned (NW), and litter weight at weaning (LWW), were determined. Sanger sequencing and PCR restriction fragment length polymorphism (PCR-RFLP) were utilized to identify the rs81471943 genotype. We found that the genotype frequency of CC was significantly higher than that of CT and TT, and CC was the most frequent genotype for NBA, LWB, NW, and LWW. Moreover, 5′-deletion and luciferase assays identified a positive transcription regulatory element in the *EXOC4* promoter. After exploring the *EXOC4* promoter, SNP −1781G/A linked with SNP rs81471943 (C/T) were identified by analysis of the transcription activity of the haplotypes, and SNP −1781 G/A may influence the potential binding of *P53*, E26 transformation specific sequence -like 1 transcription factor (*ELK1*), and myeloid zinc finger 1 (*MZF1*). These findings provide useful information for identifying a molecular marker of *EXOC4*-assisted selection in pig breeding.

## 1. Introduction

Pigs were the earliest livestock species to be domesticated. Pig breeding has a particularly long history involving artificial selection from domestication to modern breeding [1]. The abundant phenotypic variations established during pig breeding have been a valuable resource to study the economic trait mechanisms underlying domestication [2]. Causal gene identification provides useful information about the mutation types underlying the phenotypic evolution of domestic animals [3]. In porcine production, improvements in the reproductive performance of sows are slow due to the low heritability of reproductive traits [4]. The recent development of sequencing and genotyping technologies for pigs have enabled the exploration of genomic evidence of selection and the detection of candidate genes associated with target traits. Studies have found that the positive selection of pigs is associated with specific genes related to lactation [5], reproduction [6,7], meat quality [8], and growth traits [9]. Reproductive traits, such as the number of piglets born alive (NBA) [10], lactation capacity, number of piglets weaned (NW) [11], and litter weight at weaning (LWW) [12], have all been genetically improved through artificial selection.

Porcine reproduction performance is mostly evaluated by quantitative traits, which in turn are mainly affected by minor genes. These minor genes can be identified by quantitative trait loci (QTL) mapping [13]. According to PigQTLdb [14], there are 29,865 QTL associated with 688 different traits. On chromosome 18, there are three, three, two and five QTL significantly associated with porcine ovary weight [15], teat number, total offspring born alive, and corpus luteum number traits [16,17,18], respectively. In a commercial single nucleotide polymorphism (SNP) array, one SNP, rs81471943, located in the exon of the exocyst complex component 4 (*EXOC4*) gene, and six QTLs associated with reproductive traits were identified within 5–10 Mb around *EXOC4*. This suggested that *EXOC4* might be associated with reproductive traits. *EXOC4* is one of the subunits of the exocyst, which is an evolutionarily conserved complex of eight proteins, comprising EXOC1, EXOC2, EXOC3, EXOC4, and other subunits. The exocyst complex is posited to be involved in protein transfer between cells [19,20]. *EXOC4* connects vesicles to the cell membrane and participates in vesicular-mediated transport. *EXOC4* has been reported to be expressed in many human tissues, with slightly higher expression in the ovary, skeletal muscle, spleen, and hypothalamus [21]. A study investigating the gene interactions for body mass index (BMI) in a European–American adult female cohort used genome-wide interaction analyses and pathway association analyses to show that the *EXOC4*-1q23.1 interaction was associated with BMI. In the pathway-based association analysis, the Tob1 pathway, which contributes to obesity through the mitogen-activated protein kinase (MAPK) pathway, showed the most significant association with BMI. These findings were also replicated in different human populations [22]. In addition, we analyzed the general linkage disequilibrium (LD) within +/−100 Kb around the *EXOC4* gene via Haploview. However, the underlying relationship between SNP rs81471943 and reproductive traits, as well as the transcription mechanism of *EXOC4*, are still unclear in pigs.

To explore the relationship between SNP rs81471943 and reproduction traits in pigs, we acquired the genotype frequencies of SNP rs81471943 in Duroc pigs. The associations between SNP rs81471943 and reproductive traits such as NW, LWW, NBA, and litter weight at birth (LWB) were determined. Then, 5′-deletion and a luciferase assay were utilized to identify whether SNP rs81471943 was associated with the transcription of *EXOC4*. This study contributes to the understanding of the regulatory mechanisms of the *EXOC4* gene and molecular marker-assisted selection in pig breeding.

## 2. Materials and Methods

### 2.1. Ethics Approval and Consent to Participate

Experiments and animal care in this study were conducted according to the Regulations for the Administration of Affairs Concerning Experimental Animals (Ministry of Science and Technology, Beijing, China, revised June 2004) and approved by the Animal Care and Use Committee of the South China Agricultural University, Guangzhou, China (approval number: SYXK 2019-0136).

### 2.2. Animals

Ear samples from 994 Duroc sows prepared for SNP genotyping were obtained from a breeding herd and were collected from 2009 to 2017 in Fujian, China. The ear samples were collected into 75% alcohol and stored at −20 °C. TaKaRa MiniBEST Universal Genomic DNA Extraction Kit Version 4.0 (TaKaRa, Dalian, China) was utilized to extract the genomic DNA from porcine ear tissues. The A260/280 ratios of all DNA samples were determined with a NanoDrop One (Thermo Fisher Scientific, Waltham, MA, USA). All DNA samples passed A260/280 ratio (1.7–2.0) detection and were genotyped on an Illumina PorcineSNP60 BeadChip (Illumina, San Diego, CA, USA). Four reproductive traits, including NBA, LWB, NW and LWW, were recorded. NBA and LWB were measured 24 h after delivery, and LWW and NW were recorded after weaning.

### 2.3. Polymorphism Identification and Genotype with PCR-Restriction PCR-RFLP

To determine the polymorphic locus rs81471943 (C/T) of the porcine *EXOC4* gene, 8 Duroc pigs were sequenced by Sanger sequencing, and 10 Duroc pigs were genotyped by PCR-restriction fragment length polymorphism (PCR-RFLP) as confirmation. DNA extracted from the ear samples of 10 pigs was set as the template. The fragment containing SNP rs81471943 (position: 16,079,412 of chromosome 18) of *EXOC4*, a gene with a length of 640 bp (position: 16,078,898–16,079,537 of chromosome 18), was amplified using PrimerSTAR^®^ high fidelity enzyme (TaKaRa, Dalian, China). The primers are listed in Table 1 and named EXOC4-SNP. The PCR reaction system (10 μL) included: 5 μL Primer STAR mix, 0.3 μL forward primer, 0.3 μL reverse primer, 1 μL DNA and 3.4 μL double distilled H_2_O (ddH_2_O). The PCR procedure involved: pre-denaturation at 98 °C for 2 min, 35 cycles (denaturation at 98 °C for 10 s, annealing at 53 °C for 15 s, extension at 72 °C for 1 kb * min^−1^), and extension at 72 °C for 10 min.

Consequently, PCR products were separated by agarose gel electrophoresis and isolated using a Gel extraction kit (Meiji, Guangzhou, China). The isolated products were digested by *Bsr*B I (identification sequence: CCGCTC) restriction endonuclease (NEB, Ipswich, UK) and separated using agarose gel electrophoresis. PCR products with two digested fragments were CC genotype (565 bp + 75 bp), those with three digested fragments were CT genotype (640 bp + 565 bp + 75bp), and those with a single digested fragment were TT genotype (640 bp).

### 2.4. Culture of Porcine Granulosa Cells (GCs) in Vitro

GCs were cultured according to previous studies [23]. Porcine ovaries were collected from a local slaughterhouse in Guangzhou, China. The ovaries were stored in phosphate-buffered saline (PBS) containing penicillin (100 IU/mL) and streptomycin (100 μg/mL; Invitrogen, Shanghai, China) at 37 °C and transported to the laboratory quickly. Then, 5–7 mm follicles were punctured, and GCs in follicular fluid were collected using a 1 mL syringe. After washing the isolated GCs twice with PBS, the GCs were seeded into 75 cm^2^ flasks and cultured at 37 °C under 5% CO_2_ in Dulbecco’s modified Eagle medium (Hyclone, Logan, UT, USA) containing 10% fetal bovine serum (100 IU/mL penicillin and 100 μg/mL streptomycin; Hyclone, Logan, UT, USA).

### 2.5. Construction of EXOC4 5′-Deletion Fragment Vectors

According to the promoter sequences of porcine *EXOC4* from release 89 of the Ensembl genome browser (accession number: ENSSSCG00000016543, position: 16,057,927–16,346,564 of chromosome 18), we inferred that there was no leader exon present in *EXOC4*. Then, we designed 10 pairs of primers to amplify the deletion fragments of the *EXOC4* promoter (Table 1). The transcription start site of the *EXOC4* gene (position: 16,346,564 of chromosome 18) was defined as +1. Thus, the longest 5′ deletion fragment, which covered a 2657 bp sequence upstream of the transcription start site and a 134 bp sequence of the first exon, was named P0 (−2657/+134), and the other fragments were named P1 (−2204/+134), P2 (−1914/+134), P3 (−1682/+134), P4 (−1323/+134), P5 (−886/+134) and P6 (−518/+134). The shorter 5′ deletion fragments were named P3A (−1826/+134), P4A (−1551/+134), and P4B (−1225/+134). The PCR product was obtained by Taq DNA polymerase (Vazyme, Piscataway, NJ, USA). The primers are listed in Table 1. The PCR reaction system (10 μL) included: 5 μ L PCR Taq mix, 0.3 μL forward primer, 0.3 μL reverse primer, 1 μL DNA, and 3.4 μL ddH_2_O. The PCR procedure involved: pre-denaturation at 94 °C for 3 min, 35 cycles (denaturation at 94 °C for 30 s, annealing at 60 °C for 30 s, extension at 72 °C for 1 kb * min^−1^), and extension at 72 °C for 10 min. PCR products were purified by gelatinization and were added “AAA” tails to ligate with PMD-18T were performed prior to Sanger sequencing (RuiBiotech, Beijing, China). The DNA sequences of PCR products were determined by DNASTAR software. Each deletion fragment digested with *Hind* III and *MIu* I was cloned into the eukaryotic expression vector pGL3-vector, which was also digested with *Hind* III and *MIu* I restriction endonucleases.

### 2.6. Luciferase Assay

According to the manufacturer’s instructions for the dual-luciferase reporter assay kit (Promega, Madison, WI, USA), we used the BioTek Synergy 2 multifunctional microplate reader for fluorescence detection (BioTek, Winooski, VT, USA). In the luciferase assay, firefly luciferase was set as the experimental reporter and renilla luciferase was set as the control. Relative luciferase activity is calculated by the ratio of the expression of firefly luciferase (560 nm) to renilla luciferase (465 nm). The ratio of expression can normalize the activity of an experimental reporter to the activity of an internal control, which minimizes or sometimes even eliminates the experimental variability.

### 2.7. Identification of SNP and Transcription Binding Sites

For sequencing and SNP identification, 35 pigs were randomly selected from the 994 pigs used in this study. PCR was performed using PrimerSTAR^®^ high fidelity enzyme (TaKaRa, Dalian, China) to obtain the fragment containing the *EXOC4* promotor, and the primers used are listed in Table 1 (named EXOC4-Promoter). The reaction system and PCR procedure were the same as in Section 2.3. Then, the PCR product was linked to the T-vector and sequenced by Sanger sequencing (RuiBiotech, Beijing, China). After comparing with the sequences published on the Ensembl genome browser (release 89), one SNP was identified and located on −1781G/A of *EXOC4*, and this SNP also located on the potential transcription factor-binding fragment P3A-P4A (−1826/−1551) of *EXOC4*.

**Figure 1:**
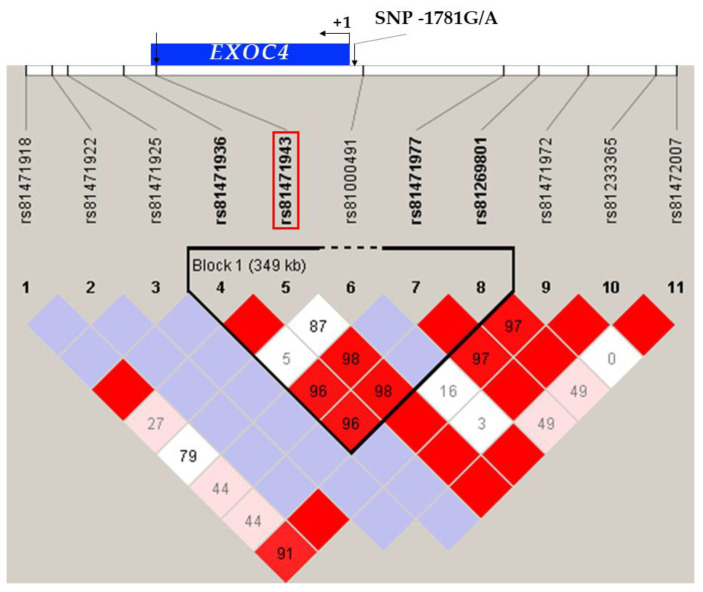
Schematic diagram of the *EXOC4* structure. Notes: The linkage disequilibrium (LD) plot within +/−100 Kb of the EXOC4 gene was computed by Haploview. The color of each square from light to dark (white to red) indicates the degree of LD from low to high.

To detect the linkage between SNP −1781G/A and SNP rs81471943 of *EXOC4*, four haplotypes were identified and defined as HA-1 (GC), HA-2 (AC), HA-3 (GT) and HA-4 (AT). The 5′ deletion fragments EXOC4-P2 (−1914/+134) of four haplotypes were amplified and cloned into the eukaryotic expression vector pGL3-vector. Luciferase assays were used to detect the effect of different haplotypes on the transcription activity of *EXOC4*. We analyzed the general linkage disequilibrium (LD) within +/−100 Kb of the *EXOC4* gene via Haploview, and the location diagram of the SNP, exons, deletion, binding sites, and promoter is shown in Figure 1. Then, the potential transcription factor binding region was also predicted by TFBIND [21] and Jaspar [22]. The results of the potential transcription factor binding site were scored by TFBIND.

### 2.8. Statistical Analysis

Estimated breeding values (EBVs) of all pigs were computed using animal model best linear unbiased prediction [24] and obtained from the in-farm genetic evaluation software platform Herdsman swine management (S & S Programming, Lafayette, IN, USA). The models used were as follows:

Y=Xb+Za+Wpe+e

where Y is a vector of phenotypic records, b is the vector of fixed effects (including parity and year-season), a is the vector of additive genes, pe is the vector of permanent environmental effects, e is a vector of residuals, and X, Z and W are incidence matrices for b, a, and pe.

The EBVs of reproductive traits (NBA, LWB, LWW and NW) were used for the multiple comparisons of SNP genotype rs81471943 by the Tukey–Kramer multiple comparison via the 9.2 version of SAS software (North Carolina State University, Raleigh, NC, USA). *p* < 0.05 indicates significant differences.

The luciferase assay was repeated at least three times independently, and the data are shown as the mean ± standard deviation (SD) of repeated experiments. The significance of differences in the means between two groups was analyzed using Student’s *t*-test (two-tailed). ** indicates significant difference at *p* < 0.01, and * indicates significant difference at *p* < 0.05.

## 3. Results

### 3.1. Polymorphisms of SNP rs81471943

The genotype frequency of the rs81471943 on the *EXOC4* gene in the 994 Duroc pigs was calculated (Table 2). Three genotypes for SNP rs81471943 were found. CC was the most frequent genotype, with a genotype frequency of 0.715, which was higher than that of CT (0.258) and TT (0.027). The most frequent allele was C, with an allele frequency of 0.844, which was higher than that of T (0.156). The χ^2^ valued 0.224 < 5.99 and confirmed that the frequency distribution of SNP rs81471943 was in accordance with the Hardy–Weinberg equilibrium law in the selected Duroc pig population.

### 3.2. Association Between SNP rs81471943 and Reproduction Traits

**Figure 2:**
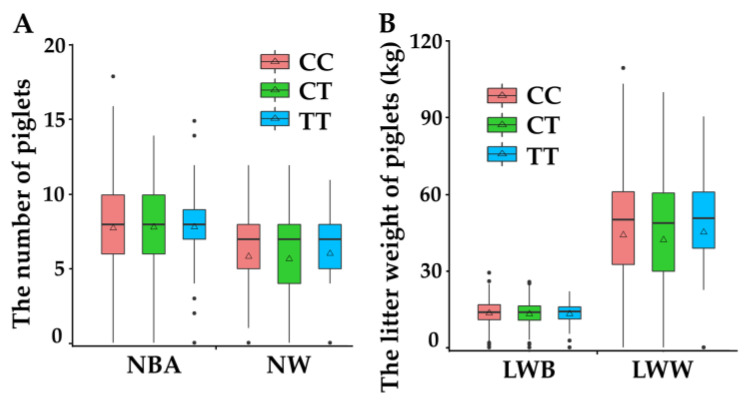
Descriptive statistics for phenotypes of Duroc pigs with three genotypes. (**A**) The descriptive statistics for genotypes of NBA and NW of the three phenotypes of Duroc pigs; (**B**) The descriptive statistics for genotypes of LWB and LWW of the three phenotypes of Duroc pigs. Notes: NBA—number of piglets born alive, LWB—litter weight at birth, LWW—litter weight at weaning, NW—number of piglets weaned.

Descriptive statistics for the phenotype of 994 Duroc sows with CC, CT and TT genotypes are shown in Figure 2. The NBA (Figure 2A), NW (Figure 2A) and LWW (Figure 2B) of individuals with CC was higher than CT and TT. The LWB (Figure 2B) of individuals with CC was higher than those with CT and lower than those with TT (Figure 2).

Moreover, descriptive statistics for EBVs of phenotypes are shown in Table 3. The EBVs of NBA of individuals with CC was higher than CT and TT, but there was no significant difference among the three genotypes. The EBVs of LWB of individuals with CC was higher than CT and lower than TT. The EBVs of NW and LWW of individuals with CC was significantly higher than CT and non-significantly higher than TT (Table 3). These observations suggested that CC was the most frequent genotype for NW and LWW.

### 3.3. Isolation of SNP rs81471943 on EXOC4

**Figure 3:**
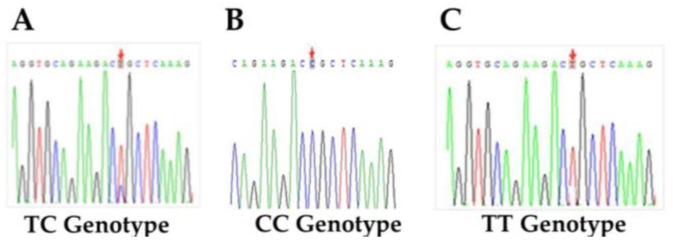
Sanger sequencing of SNP rs81471943 (C/T) on *EXOC4.* SNP rs81471943 (C/T) polymorphism locus is located at position 16,079,412 of chromosome 18, and the three genotypes were CC (**A**), CT (**B**), and TT (**C**), respectively. M1000: DNA marker of 1000 bp.

**Figure 4:**
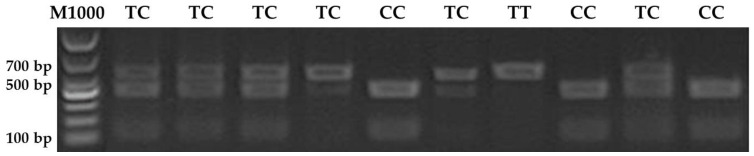
PCR-restriction fragment length polymorphism (RFLP) detection of rs81471943 in Duroc pigs. M1000: DNA marker of 1000 bp. Three genotypes were identified by *Bsr*B I. PCR products with two digested fragments were CC genotype (565 bp + 75 bp); PCR products with three digested fragments were CT genotype (640 bp + 565 bp + 75 bp); and PCR products with one digested fragment were TT genotype (640 bp).

Target fragments of *EXOC4* containing SNP rs81471943 were amplified from extracted DNA from eight Duroc pigs and identified by Sanger sequencing (Figure 3A–C). Compared with the sequence of *EXOC4* in the Ensembl genome browser (release 89), a polymorphic mutation base C/T was found on *EXOC4* (position: 16,079,412 of chromosome 18), which is in line with SNP rs81471943 in the commercial SNP array. To determine the polymorphic loci SNP rs81471943 of *EXOC4*, 10 pigs were used in the PCR-RFLP detection. As shown in Figure 4, the three genotypes CC, CT and TT were identified by restriction endonuclease *Bsr*B I.

### 3.4. Transcription Activity Analysis of the EXOC4 Promoter

**Figure 5:**
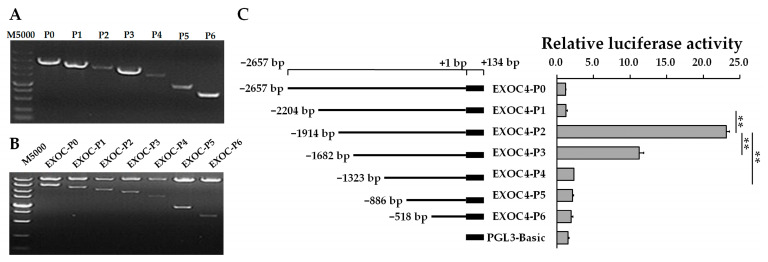
Transcription activity of the 5′ deletion fragment of the *EXOC4* promoter. (**A**) PCR products of 5′ deletion fragments from *EXOC4* promoter; (**B**) enzyme digestion identification of pGL3-vector with a deletion fragment of the *EXOC4* promoter by restriction enzyme digestion; (**C**) the relative luciferase activity of the 5’ deletion fragment on the *EXOC4* promoter. M5000: DNA marker of 5000 bp. ** indicates *p* < 0.01. Data are presented as means ± SD.

To identify regulatory elements on the *EXOC4* promoter, 5′-deletion and luciferase assays were used. Six fragments with a 5′-deletion of the *EXOC4* promoter were amplified (Figure 5A) and cloned into pGL3-vector (Figure 5B). Compared with EXOC4-P2 (−1914/+134), the relative luciferase activity of EXOC4-P1 (−2204/+134), EXOC4-P3 (−1682/+134), and EXOC4-P4 (−1323/+134) were all significantly decreased (Figure 5C). This indicated that the P1–P2 (−2204/−1914) region might harbor the negative control elements, and the P2–P4 (−1914/−1323) region might harbor the positive transcription regulatory elements.

**Figure 6:**
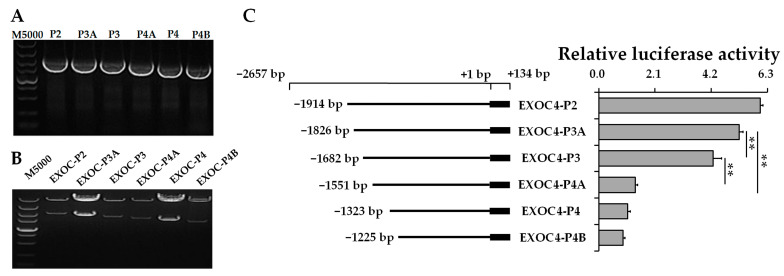
Transcription activity of the P2–P4 region of *EXOC4*. (**A**) PCR products of smaller 5′ deletion fragments from the promoter of *EXOC4*; (**B**) enzyme digestion identification of pGL3-vector with a further deletion fragment of the *EXOC4* promoter; (**C**) the relative luciferase activity of a smaller 5’ deletion fragment on the *EXOC4* promoter. M5000: DNA marker of 5000 bp. ** indicates *p* < 0.01. Data are presented as means ± SD.

Then, the shorter 5′ deletion fragments of P3A (−1826/+134), P4A (−1551/+134), and P4B (−1225/+134) were amplified (Figure 6A) and cloned into the eukaryotic expression vector pGL3-vector (Figure 6B). Compared with EXOC4-P3A, the relative luciferase activity of EXOC4-P3 and EXOC4-P4A were significantly decreased. Similarly, compared with EXOC4-P3, the relative luciferase activity of EXOC4-P4A was significantly decreased (Figure 6C). These results indicated that positive transcription regulatory elements might exist in the P3A–P4A (−1826/−1551) region of *EXOC4*.

### 3.5. Transcription Activity Analysis of Different Haplotypes of the EXOC4 Gene

The −1826/−1551 fragment of *EXOC4* was amplified and sequenced. Interestingly, one SNP was identified as being located on −1781 of *EXOC4*. As shown in Table 4, there was an SNP located on −1781G/A of *EXOC4*, and four haplotypes were defined as HA-1 (GC), HA-2 (AC), HA-3 (GT), and HA-4 (AT).

**Figure 7:**
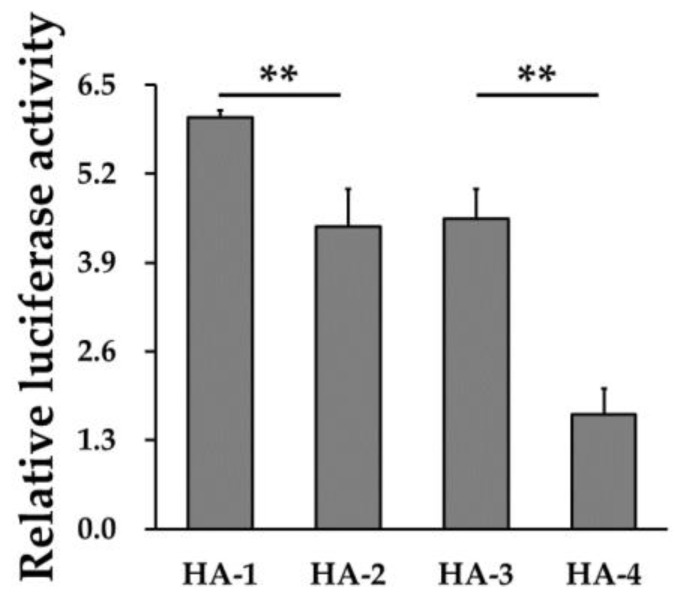
Transcriptional activity of different haplotypes at the binding region of *EXOC4*. The relative luciferase activity of *EXOC4* with different haplotypes (HA-1 (GC), HA-2 (AC), HA-3 (GT), and HA-4 (GT)). ** indicates *p* < 0.01. Data are presented as means ± SD.

As shown in Figure 7, we found that the luciferase activity of HA-1 was significantly (*p* < 0.05) higher than HA-2, whilst HA-3 was significantly (*p* < 0.05) higher than HA-4. These observations suggested that the SNP rs81471943 might link with −1781G/A and −1781G to potentially affect the expression of *EXOC4*. Moreover, many potential binding sites of transcription factors were predicted on −1781G/A of *EXOC4* (Table 5). *P53* and ETS transcription factor (*ELK1*) might bind at −1781A, while myeloid zinc finger 1 (*MZF1*) might bind at −1781G.

## 4. Discussion

The exocyst is a protein complex composed of *EXOC1* to *EXOC8*. The exocyst mediates the docking of vesicles carrying membrane proteins [25]. *EXOC4* is a component of the exocyst complex, which is associated with various phenomena such as cell migration, endophoria formation, cytokinesis, glucose uptake, and neural development in mammals. Previous studies indicate that the mutation of specific SNP loci in *EXOC4* leads to an increase in the malformation rate of human newborns [26]. *EXOC4* is also involved in insulin, triiodothyronine, and thyroxine secretion in Chinese Holstein cattle [27]. Jiao et al. investigated the interactions between *EXOC4*-1q23.1 and BMI in a European–American adult female cohort via genome-wide interaction analyses, and results suggest that *EXOC4*-related pathways may contribute to the development of obesity [22]. Similarly, after *EXOC4* knockout in embryos, mice can form gastrointestinal embryos normally, but are unable to progress beyond the primitive streak stage and die shortly after [20]. In addition, genome-wide association studies in chickens also report that three QTL are located on *EXOC4* and are associated with growth traits of 49–56 day old chickens [28], bodyweight of 63 day old chickens [29], and pectoralis weight of 70 day old chickens [30], respectively. These observations suggest that *ECOX4* might be important for economic traits in livestock.

In this study, we found the SNP rs81471943 (C/T) located on the *EXOC4* gene. After analyzing the relationship between genotype of the *EXOC4* and reproductive traits, three genotypes (CC, CT and TT) were found in the Duroc pig population, while C was the most frequent allele. Moreover, multiple comparisons between SNP and phenotypes confirmed that CC was the most frequent genotype on NW and LWW in Duroc pigs (Figure 2 and Table 3). Previous studies have shown that there are three QTL which are significantly associated with teat number [16,17,18] on chromosome 18, which indicated that *EXOC4* could affect the lactation performance of commercial pigs. In this study, we also found that CC was significantly associated with NW and LWW, but not NBA or LWB. In addition, previous studies demonstrated that reproductive traits were highly correlated with each other, such as NBA and LWB [31]. In this study, we found that CC was the most frequent genotype for NBA, but not LWB. This observation might be caused by the limited sample size used in this study, and it is likely that CC would be significantly associated with NBA in a larger population size [32]. Collectively, although the results were based on a relatively small population, to some extent, the association of SNP rs81471943 and lactation capacity, NW, and LWW could provide useful information for *EXOC4*-mediated reproduction in pigs.

It is well known that transcription factors modulate gene expression by interacting with the promoter regions of related genes. For example, p65 may target the −348/−338 region of fibroblast growth factor receptor 1 (*FGFR1*) to promote the transcription of *FGFR1* and enhance the pro-proliferation and anti-apoptotic effect of *FGFR1* to facilitate the growth of follicles [33]. Thus, to explore effects of rs81471943 on the expression of *EXOC4*, the SNP markers which link with rs81471943 at the promoter of *EXOC4* were further investigated by constructing 5′-deletions for *EXOC4*. Interestingly, we found that positive regulatory elements might localize in the P3A–P4A (−1826/−1551) region (Figure 6), and the negative control element might localize in the P1–P2 (−2204/−1914) region (Figure 5). After exploring the SNPs in the P3A–P4A (−1826−1551) region (containing positive transcription regulatory elements), one SNP was found to be located on −1781. Then, we further analyzed the transcription activity of the four haplotypes. The results showed that SNP rs81471943 might link with SNP −1781G/A, and SNP −1781G showed the potential to affect the expression of *EXOC4* (Figure 7). These results indicate that SNP rs81471943 might link with SNP −1781G to affect the expression of *EXOC4*.

The literature indicates that −1781G/A might regulate the expression of *EXOC4* by affecting the binding of transcription factors. In this study, we also predicted that the transcription factors *P53*, *ELK1*, and *MZF1* would be located on the −1826/−1551 region of the porcine *EXOC4* promoter. *P53* participates in maintaining cell growth [34]. *P53* can enhance stability through phosphorylation and the activation or inhibition of the transcription of downstream genes, thus inducing cell cycle arrest and apoptosis [35]. *ELK1* is a transcription factor belonging to the ETS oncogene family and induces endothelial-to-myofibroblast transition of tumor endothelial cells [36], which plays an important role in breast and ovarian cancers [15]. As a bifunctional transcription factor, *MZF1* belongs to the zinc finger protein Kruppel transcription factor family, which regulate downstream gene expression by binding to the cis-acting element TGGGGA on gene promoters [37,38]. Results in Table 4 and Figure 7 indicate that the mutation of −1781G might be the cause of the differences in *EXOC4* transcription. Taken together, SNP rs81471943, which was significantly associated with NW and LWW, might link with SNP −1781G/A, localizing at the positive regulatory elements of the *EXOC4* promoter, to affect the expression of *EXOC4* in Duroc pigs.

## 5. Conclusions

In Duroc pigs, we found that the CC genotype frequency was significantly higher than CT and TT in SNP rs81471943, and CC was the most frequent genotype for NW (*p* = 0.01) and LWW (*p* < 0.01). The −1826/−1551 region of *EXOC4* contains a positive regulatory element, and the haplotypes of SNP −1781G/A and SNP rs81471943 might significantly affect the transcription activity of *EXOC4*. Moreover, SNP −1781G/A might influence the binding of the potential cis-acting elements *P53*, *ELK1* and/or *MZF1*. These findings reveal that SNP rs81471943 is significantly associated with the reproductive traits NW and LWW in Duroc sows.

## Figures and Tables

**Table 1:** Primers used in this study. SNP: single nucleotide polymorphism.

Name	Primer Sequence	Fragment Length (bp)
EXOC4-SNP	F: ACAGCCTCGGCTCAACCTTA	640
	R: TGCTTTTACGAAGGGGGACA	
EXOC4-Promoter	F: GAGCGAGTCCTTGTCTACAGT	2791
	R: TGCTTTTACGAAGGGGGACA	
P0 (−2657/+134)	F: CGACGCGTGAGCGAGTCCTTGTCTACAGT	2791
	R: CCCAAGCTTGCGCATTGGGGATTCTTACA	
P1 (−2204/+134)	F: CGACGCGTGGGAACTCGTTCTTTCCCCC	2338
P2 (−1914/+134)	F: CGACGCGTCCACTCGGACTGTCATCAGC	2048
P3 (−1682/+134)	F: CGACGCGTAAGATGGGAGATGGTTCGGG	1816
P4 (−1323/+134)	F: CGACGCGTGGATGGCTGGATTCCACACT	1457
P5 (−886/+134)	F: CGACGCGTTTTTACAGGTTACTGGTCAGACT	1020
P6 (−518/+134)	F: CGACGCGTATTTAATGTGCAGGACCATGCG	652
P3A (−1826/+134)	F: CGACGCGTATGGCTATGTCTAGCCTCCC	1950
	R: CCCAAGCTTGCGCATTGGGGATTCTTACA	
P4A (−1551/+134)	F: CGACGCGTACCAGCCACCTCACTTTTGA	1685
P4B (−1225/+134)	F: CGACGCGTTCATTGGGATGCTACCAGGC	1359

**Table 2:** Genotype and allele frequency of SNP rs81471943 in the *EXOC4* gene in Duroc pigs.

Genotype	Sample Quantity	Genotype Frequency	Allele	Allele Frequency	χ^2^
CC	711	0.715	C	0.844	0.224
CT	256	0.258	T	0.156	
TT	27	0.027			

χ^2^ 0.05 (2) = 5.99.

**Table 3:** Multiple comparisons between genotypes of SNP rs81471943 and estimated breeding values (EBVs) of reproductive traits in Duroc pigs.

Genotype	Genotype Frequency (Number)	NBA EBV	LWB EBV	NW EBV	LWW EBV
CC	0.715 (711)	0.21 ± 0.19 ^a^	0.24 ± 0.31 ^ab^	0.61 ± 0.26 ^a^	6.06 ± 1.94 ^a^
CT	0.258 (256)	0.17 ± 0.09 ^a^	0.12 ± 0.14 ^a^	0.36 ± 0.20 ^b^	3.66 ± 1.48 ^b^
TT	0.027 (27)	0.12 ± 0.07 ^a^	0.38 ± 0.12 ^b^	0.53 ± 0.19 ^ab^	5.35 ± 1.43 ^ab^

Notes: NBA—number of piglets born alive, LWB—litter weight at birth, LWW—litter weight at weaning, NW—number of piglets weaned. The multiple comparisons between the SNP genotype and EBVs of NBA, LWB, NW and LWW were calculated using the Tukey–Kramer method. The data used were the EBVs of least square means (LSM) ± standard errors (SE). The ^a, b, ab^ were multiple comparisons result of Tukey test. In same column, LSM ± SE of EBVs followed by different lowercase letters indicated significant difference (*p* < 0.05) and followed by same lowercase letters indicated no significant difference (*p* > 0.05).

**Table 4:** Relationship between Duroc pigs of different *EXOC4* genotype with the SNP promoter.

Haplotype	HA-1	HA-2	HA-3	HA-4
−1781	G	A	G	A
rs81471943	C	C	T	T

**Table 5:** Prediction of potential binding sites on −1781G/A.

TF	Nucleotide Location	Chain	Scored	Position	Sequence Pattern
*P53*	−1786–1777	−	0.796	−1781A	AGGA**A**GGTCA
*ELK1*	−1785–1773	−	0.783	−1781A	ACGTGAGGA**A**GGTC
*MZF1*	−1787–1775	+	0.856	−1781G	TGAGGA**G**GGTCAT

Note: The mutation site is in bold.

## Data Availability

The data presented in this study are available on request from the corresponding author.
